# Foundational Research Could Improve Future Transcutaneous Electrical Nerve Stimulation Evaluations

**DOI:** 10.3390/medicina58020149

**Published:** 2022-01-19

**Authors:** Peter W. Gladwell, Fiona Cramp, Shea Palmer

**Affiliations:** 1 Pain Management Service, North Bristol NHS Trust, Bristol BS10 5NB, UK; 2 Faculty of Health and Applied Sciences, University of the West of England, Bristol BS16 1QY, UK; Fiona.Cramp@uwe.ac.uk; 3 Centre for Care Excellence, Coventry University and University Hospitals Coventry and Warwickshire NHS Trust Richard Crossman Building, Jordan Well, Coventry CV1 5RW, UK; ad6948@coventry.ac.uk

**Keywords:** transcutaneous electrical nerve stimulation, musculoskeletal pain, low back pain, patient reported outcome measures, qualitative research, complex intervention

## Abstract

*Background and objectives:* There is a lack of good quality evidence regarding the effectiveness of transcutaneous electrical nerve stimulation (TENS) for chronic musculoskeletal pain, including chronic low back pain. High quality randomised controlled trials (RCTs) have been called for to establish effectiveness over and above placebo and some guidance has already been offered regarding the design of such trials. This article expands the discussion regarding the design of future TENS trials. There is qualitative evidence of the complexity of TENS as an intervention which should be considered in future TENS evaluations. This complexity includes multiple benefits reported by patients, depending on their chosen contexts of TENS use. The ideal content and delivery of support for patients to optimise TENS use also lacks consensus. There is no evidence that a TENS education package has been designed to support the complex set of behaviours and choices which experienced users suggest are required to optimise TENS benefits. Finally, clinical and research outcomes have not been contextualised and related to the specific strategies of use. *Conclusions:* We suggest that research is required to develop consensus about the content and delivery of training in TENS use for patients who live with pain, informed by the experience of patients, clinicians, and researchers. Once a consensus about the content of TENS training has been reached, there is then a need to develop a TENS training course (TTC) based on this content. An effective and acceptable TTC is needed to develop the knowledge and skills required to optimise TENS use, supporting patients to build confidence in using TENS in everyday life situations with the aim of reducing the impact of chronic pain on function and quality of life. Further research is required to extend the evidence base regarding appropriate, contextualised TENS patient-reported outcomes.

## 1. Introduction

The prevalence of moderate-to-severe non-cancer chronic pain across Europe has been reported as 19% [1] with 42% of those reporting back pain. Chronic pain negatively impacts the quality of working and social lives with 19% of those affected having lost their job because of pain [1]. Low back pain is the most common and disabling musculoskeletal health problem and is the leading worldwide cause of years lost to disability [2,3]. Non-specific chronic low back pain (CLBP) causes a significant economic burden for individuals, the economy and society [4] and is associated with increased healthcare costs [5]. Chronic pain is therefore a serious problem, and its impact indicates that current pain treatments have limited benefits, and/or that they fail to reach those in need.

TENS is a low voltage electrical stimulation aiming to relieve pain by activating sensory nerves. It can also be used to stimulate motor nerves when it is known as “acupuncture-like” TENS [6]. TENS is generated using a portable, battery-powered device, with stimulation delivered via electrodes attached to the skin. TENS devices are widely available via pharmacies and internet-based suppliers and can be administered by a patient, therapist, or carer. Qualitative evidence indicates that TENS can be integrated into a patient’s self-management to support independence, and that TENS use can be medication-sparing [7,8]. A typical cost of under £30 for a TENS unit compares favourably against the cost to healthcare systems and to patients of repeat medication prescriptions. There are significant risks from pain medications and limited benefits [9] and in this context TENS has the potential to offer inexpensive, drug-free pain relief with minimal side effects [10] and some contraindications [6]. People with persistent pain have reported that they find TENS helpful [7,8,10]. In 2006, Breivik [1] reported that only 5% of their European survey respondents had tried TENS or a similar form of nerve stimulation: there has been no large-scale survey which has updated this finding. The purpose of this review paper is to outline the methodological limitations of existing TENS studies and provide recommendations for future research.

## 2. Evidence of TENS Effectiveness

There is evidence to support the hypoalgesic effect of TENS from laboratory studies on humans using experimentally induced pain and from animal studies using tissue damage paradigms [11]. The largest meta-analyses on TENS for chronic musculoskeletal pain [12] and post-operative analgesic consumption [13] were positive, and TENS is recommended as an adjunct to core treatments for pain relief in the NICE guideline for osteoarthritis [14]. Two recent randomised controlled trials (RCTs) of TENS for people with fibromyalgia have demonstrated a reduction in pain at rest, movement-evoked pain and fatigue [15,16]. However, the most recent Cochrane review of TENS for CLBP [17] was inconclusive due to equivocal findings, as was a recent overview of Cochrane Reviews of TENS for chronic pain, due to methodological weakness in the original studies [18]. As a result of the lack of good quality evidence about TENS effectiveness for low back pain, the NICE guideline for low back pain and sciatica [9] recommended that TENS should not be used in the National Health Service for back pain. Despite five decades of TENS use, there remains insufficient high-quality evidence to determine whether it has benefits over and above placebo for people with chronic musculoskeletal pain or more specifically CLBP. There is a clear need for better quality evaluations of the effectiveness of TENS for chronic pain, and more specifically CLBP.

## 3. Problems with Trial Fidelity Have Been Identified

TENS is recommended as an adjunct to core treatments for pain relief by the NICE guideline for osteoarthritis [14]. There is an obvious discrepancy between the evidence base regarding osteoarthritis, and other persistent pain conditions. High quality research is required to generate knowledge about the effectiveness of this non-drug method of pain relief which can be used as an adjunct to self-management and rehabilitation for musculoskeletal pain conditions. In 2011, a review of the implementation fidelity of clinical trials of TENS for chronic pain identified a range of methodological weaknesses including lack of instruction on how to self-administer TENS devices, limited duration of TENS application, insufficient intensity, lack of assessment of adherence and limited reporting of the pattern and duration of TENS use [19]. Previous RCTs have failed to integrate evidence-based TENS training into the study design [19]. Under-dosing, due in part to a lack of adherence to regimens by patients, has been identified as a particular problem [11,19]. High quality RCTs have been called for to establish the effectiveness of TENS over and above placebo and detailed guidance has been offered regarding the design of such trials [11,20,21]. We aim to expand this design issue further, based on our qualitative research exploring the patient experience of TENS use [7,8,22,23]. There is currently no consensus regarding the optimal content and delivery of TENS training or optimal TENS use and no consensus about measuring TENS outcomes. These issues will be explored in more detail in subsequent sections. Prior to starting further evaluation, we argue that research is required to fill these knowledge gaps, as a foundation for a future pragmatic trial of TENS.

## 4. TENS Is a Complex Intervention with Varied Outcomes Which Relate to the Context of Use

There is qualitative evidence of the complexity of TENS as an intervention [7,8,22] which should be considered in any future TENS evaluation. This complexity includes multiple outcomes reported by patients depending on their chosen contexts of TENS use. In-depth qualitative research [7,8,22] has indicated that experienced TENS users make choices about their use of TENS in a focused, strategic manner to help them to cope in selected, specific contexts. These contexts include chosen activities such as managing journeys or shopping, and specific experiences such as pain exacerbations or pain-related insomnia. When these patient-reported benefits of TENS use were crossmatched against Patient Reported Outcome Measures (PROMs) used in previous TENS evaluations, a low degree of match was found. The percentage match was at best 44% against the Low Back Outcome Score and at worst 22% against the Roland and Morris Disability Questionnaire [23]. Patients who are experienced in using TENS report outcomes in several domains rather than just pain relief, and RCTs have rarely focused attention on these broader outcomes [8,23].

One example of contextualised use is to help with pain-related insomnia. Some users may only apply TENS at night to help them manage their pain to help them to fall asleep. One experienced TENS user explained:

*“…I’ve got to try and go to sleep with it on as well, so if I can’t go to sleep I will put the TENS on and go to sleep.”*

This reduction in pain-related insomnia might be overlooked in a TENS evaluation, despite it being the primary benefit for some patients, unless we enquire specifically about the context in which TENS is used.

Another example of contextualised use is during a pain exacerbation, or pain flare-up. One experienced TENS user chose not to use TENS during everyday life as she was too busy to fit the device, and made indirect reference to the psychological benefits of TENS by describing the emotional suffering which she experienced during a pain flare:

*“Yeah, they’re awful. Those days I’ve just, I have said I would rather not be there on my bad days ‘cause they are just awful. They are horrible.”*

The modest pain relief which she reported helped to ameliorate this suffering. She completed a pain Visual Analogue Scale to indicate pain severity of 100 mm during a flare (the anchor described “pain as bad as possible”). She indicated a reduction in pain severity to 84 mm as a result of TENS use. This 16% pain reduction falls into the 10–20% pain reduction indicated by IMMPACT [24] as a minimal clinically important difference. For her it is clearly enough of a difference for the use of TENS to be worthwhile. However, this benefit of her intermittent TENS use would be unlikely to be captured by a pain scale asking about average pain severity, unless we ensure a clear focus on the use of TENS during a pain flare.

## 5. What Foundational Research Is Required Prior to Further TENS Evaluation?

TENS training is often very poorly described within the literature, such that its content and delivery cannot be replicated. There are currently no national or international standards or guidelines to inform the delivery of TENS training in clinical settings, and we suggest that research is required to develop consensus about the content and delivery of training in TENS use for patients who live with pain, informed by the experience of patients, clinicians, and researchers. This consensus will inform the development of training and is needed to address fidelity issues in any future TENS evaluation to avoid research waste [18].

There is currently no evidence that a TENS education package has been designed to support the complex set of behaviours and choices which experienced users suggest are required to optimise TENS benefits. A review of the literature identified only one study that considered TENS education [25]. Only didactic education was provided with no consideration of the complex skills required to integrate TENS into pain self-management. Such a package will be important as a facilitation strategy to enhance implementation fidelity in any future TENS evaluation. As well as offering an enriched intervention for future research, consensus-based co-designed TENS training has the potential to bring more immediate benefits to patients within healthcare services where TENS is currently offered as an adjunct to treatment.

A consensus is required about several related issues:

1. Information provided to patients before they try TENS (such as contraindications, risks, and potential benefits)
2. Guidance to support the first trial of TENS (e.g., pad placement, intensity)
3. Support offered to facilitate the development of skills (behaviours) to optimise TENS use in everyday life
4. How to reinforce these skills as part of TENS training, for example modelling, goalsetting, or record keeping using a TENS practice diary.
5. Optimal follow-up support for patients, following the initial teaching session and trial.

## 6. The Consensus on TENS Training Will Be the Foundation for the Development of a TENS Training Course

Once a consensus about the content of TENS training has been reached, there is a need to develop a TENS training course (TTC) based on this content. An effective and acceptable TTC is needed to develop the knowledge and skills required to optimise TENS use, supporting patients to build confidence in using TENS in everyday life situations with the aim of reducing the impact of chronic pain on function and quality of life. The effectiveness of TENS training can be determined by evaluating its impact on a patient’s confidence to use TENS in everyday situations. Provision of effective TENS training would provide a firm foundation for the assessment of the clinical effectiveness of TENS. TENS training is normally delivered face to face, either in one-to-one clinical encounters, or in group-based training courses for patients. An alternative approach of an internet-delivered TTC has potential benefits for healthcare services, as it can be delivered at a much lower cost than multiple individual or group appointments. Internet-based training would also support the delivery of a multi-centre trial. The burden of chronic musculoskeletal pain including the cost to healthcare has been outlined above, and optimised training in TENS has the potential to reduce this burden for patients, and therefore indirectly benefit healthcare by reducing costs such as GP consultations and medication prescriptions. An online course can also improve the accessibility of the training for patients in remote localities, reducing the need for patients to travel long distances for face-to-face appointments. Patients who cannot or prefer not to access an internet-based course could be offered face to face support from a clinician, but this clinician can optimise their TENS teaching by learning from the internet-based course designed for potential TENS users.

## 7. TENS as a Complex Intervention Has Complex Outcomes: How Do We Capture the Benefits of Contextualised TENS Use?

Further research is required to extend the evidence base regarding the TENS patient-reported outcomes. Qualitative research has generated knowledge about the use and benefits of TENS reported by patients with chronic musculoskeletal pain [7,8,22]. Examples of patient reported benefits that extended beyond pain relief include the achievement of functional goals, coping with pain exacerbations, enhancing rest periods, managing pain-related insomnia, and reducing pain medications. This work concluded that TENS should be considered as a complex intervention as it is an intervention with “several interacting components” [26]. The dimensions of complexity which relate to TENS are the “number and variability of outcomes”, the “variability of the outcomes in the target population”, the “number and difficulty of behaviours required by those delivering or receiving the intervention” and the “degree of flexibility or tailoring of the intervention permitted”. The qualitative research relating to the patient-reported benefits of TENS has informed the development of a process evaluation [26] which goes beyond the basic competence in setting up the TENS unit and emphasises the importance of building confidence in TENS use in real life contexts. This indicates that clinical and research outcomes need to be contextualised and related to the specific strategy of use. Previous failure to do this might have partially contributed to the inconsistent evidence on effectiveness. Instead of designing research which asks what TENS does to patients as passive recipients of a treatment, we may benefit from focussing evaluation on what patients as active agents choose to do with TENS.

## 8. A Large-Scale Survey Could Expand Our Knowledge of the Patient-Reported Benefits of TENS Use

The findings of the in-depth qualitative work might not be representative of a wider population of TENS users, but a large-scale survey would expand our knowledge of the patient-reported benefits of TENS. The survey could be designed to generate data about the frequency of context-based, strategic use of TENS amongst this wider population and the survey questions could be informed by the existing in-depth qualitative research findings [7,8]. Data could be collected about functional activities which are and are not helped by TENS. This is important, as there is some evidence that the use of TENS to support function is dependent upon the range of movement and speed of movement required to perform activities. These activities are influenced by the problems associated with TENS use [22], so that more sedentary and less dynamic activities may be more likely to be chosen by patients when they are deciding which activities to support with TENS. For example, activities involving sitting such as travelling and social activities may be more commonly supported by TENS than bending, lifting, running and sports. The survey will enquire about patient-reported benefits already identified through the in-depth qualitative research, and free text sections would allow respondents to report any other activities which they support by using TENS. The dataset generated could be crossmatched against potential PROMs to inform selection for a future TENS evaluation, to ensure that the trial outcomes reflect patient experiences.

## 9. Overall Summary

In summary, there is a lack of good quality evidence regarding the effectiveness of TENS for managing chronic pain and CLBP. TENS is a complex intervention with patient-reported outcomes dependent on their chosen context of use. There is no consensus regarding the optimal delivery of TENS training in a clinical or research setting. We argue that a broader research programme is required that will carry out the foundational research required to address these issues.
